# Empirically classifying network mechanisms

**DOI:** 10.1038/s41598-021-99251-7

**Published:** 2021-10-15

**Authors:** Ryan E. Langendorf, Matthew G. Burgess

**Affiliations:** 1 Cooperative Institute for Research in Environmental Sciences, University of Colorado Boulder, 216 UCB, Boulder, CO 80309 USA; 2 Environmental Studies Program, University of Colorado Boulder, 397 UCB, Boulder, CO 80303 USA; 3 Department of Economics, University of Colorado Boulder, 256 UCB, Boulder, CO 80309 USA

**Keywords:** Computational science, Systems analysis, Machine learning, Software

## Abstract

Network data are often explained by assuming a generating mechanism and estimating related parameters. Without a way to test the relevance of assumed mechanisms, conclusions from such models may be misleading. Here we introduce a simple empirical approach to mechanistically classify arbitrary network data as originating from any of a set of candidate mechanisms or none of them. We tested our approach on simulated data from five of the most widely studied network mechanisms, and found it to be highly accurate. We then tested 1284 empirical networks spanning 17 different kinds of systems against these five widely studied mechanisms. We found that 387 (30%) of these empirical networks were classified as unlike any of the mechanisms, and only 1% or fewer of the networks classified as each of the mechanisms for which our approach was most sensitive. Based on this, we use Bayes’ theorem to show that most of the 70% of empirical networks our approach classified as a mechanism could be false positives, because of the high sensitivity required of a test to detect rarely occurring mechanisms. Thus, it is possible that very few of our empirical networks are described by any of these five widely studied mechanisms. Additionally, 93 networks (7%) were classified as plausibly being governed by each of multiple mechanisms. This raises the possibility that some systems are governed by mixtures of mechanisms. We show that mixtures are often unidentifiable because different mixtures can produce structurally equivalent networks, but that we can still accurately predict out-of-sample functional properties.

## Introduction

Interventions in complex systems and forecasts of their behavior are most likely to succeed under novel conditions when they are based on mechanistic explanations^1^. Network data and models describing complex systems have become legion, but there are still relatively few methods for discovering governing mechanisms from network data with statistical tests^2^ or machine learning^3^. Empirically understanding how networks function is increasingly important as scientists are being asked to develop systemic interventions ranging from drugs that alter cellular machinery^4^ to management plans for ecosystems in a changing climate^5^ to more just social infrastructure^6^.

Empirical network studies often assume a particular governing mechanism (model) and then estimate mechanism-specific parameters. For example, Barabási and Albert^7^ famously found that websites link to each other on the internet according to the preferential attachment mechanism with an attachment power of 2.1. The result is an unequally accessible internet where new websites are exponentially less likely to be linked to and discovered. However, alternative mechanisms are rarely considered in such studies despite evidence that multiple mechanisms can produce structurally similar networks^2,8,9^. Presuming a mechanism for network data enables powerful insights but introduces potential for tautological conclusions. Network scientists can avoid this issue by non-parametrically correlating properties of networks^10^ or individual nodes^4^ with important outcomes like stability or persistence, but at the cost of understanding the mechanistic nature of these associations which is critical for effective intervention and prediction.

Here, we introduce a general approach to empirically classify network mechanisms. By comparing an unknown network to networks simulated from known mechanisms we can—with high sensitivity and specificity—classify any empirical network as either resulting from any of a candidate set of mechanisms, or being the product of none of them.

## Network comparisons distinguish mechanisms

**Table 1:**
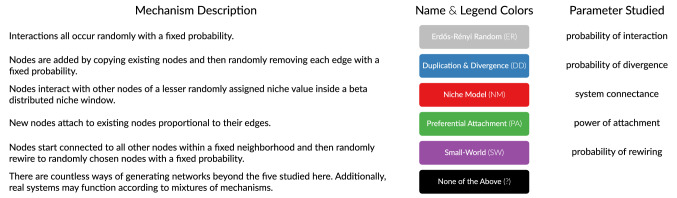
The five network generating mechanisms we considered.

For each, the **Parameter Studied** is the what the classifier systematically varies in its search for a version of each proposed mechanism that best describes the network being classified. Though we only considered one parameter per mechanism, some mechanisms have more than one governing parameter (e.g. SW’s neighborhood size), which likely means our estimates of mechanism identifiability are conservative. For canoncial references see ER^13^, DD^14^, NM^15^, PA^7^, and SW^16^. Our approach is not limited to these five mechanisms. Note that the color behind each mechanism’s name is the color used to refer to that mechanism throughout the paper.

Our method, which is available in the open-source R package *netcom*^11,12^, is a comparative approach to network classification. It systematically compares a network of interest to networks simulated from candidate mechanisms. Here we construct these candidate networks by growing networks where nodes attach to each other according to the rules of one of the five mechanisms listed and defined in Table 1: Erdös-Rényi random^13^, Duplication and Divergence^14^, the Niche Model^15^, scale-free Preferential Attachment^7^, and Small-World networks^16^. Our approach allows us to test if a network came from any of these five mechanisms, and can readily incorporate any other mechanism that can be simulated.

We use a stacking ensemble approach to capture the many ways network structures differ across mechanisms, combining 9 network properties into a measure of how different two networks are: in and out degree distributions, entropy of in and out degree distributions, clustering coefficient (transitivity), the distribution of Google’s PageRank across nodes^17^, the number of communities^18^, and the numbers of each possible 3-node and 4-node motifs. Additionally, we recalculate each of these on a fifth-order row-normalized Markov version of each network to include indirect effects. We combine (stack) these 18 network properties into a single number measuring the difference $d \left( N_i, N_j \right)$ between two networks $N_i$ and $N_j$. To do this, we measure the Euclidean distance between the values of each property in the two networks, and calculate the average across all 18 properties weighted by each property’s loading in the first axis of a principal components analysis (PCA) of networks simulated systematically across all candidate mechanisms (Table S1). Note that correlations between network properties are accounted for in so much as their eigenvectors will be correlated in the PCA and reflected in the resulting weights.

**Figure 1:**
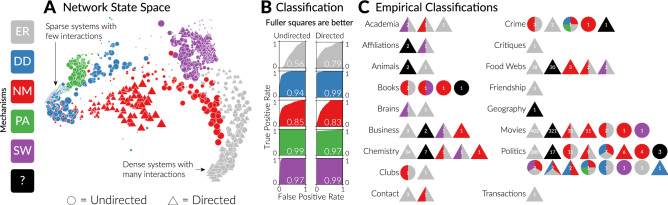
A *Network State Space* was made from the 3000 simulated networks (1500 directed and undirected each, across the five mechanisms, each with 20 nodes) by pairwise comparing 18 properties of each network in a stacking ensemble method. A two-dimensional NMDS projection (Nonmetric Multidimensional Scaling ordination, calculated using the metaMDS function in the R package *vegan*^25^) of this state space (with identical axis scaling) shows that networks cluster by mechanism. In this plot points are each a network, with their radius proportional to the governing parameter. Circles are undirected networks and triangles are directed networks. *Classification* ROC curves and corresponding AUC values (inset numbers) for the ability to classify these 3000 networks. When AUC = 1 the classifier can identify all true positives without including any false positives. Random classifiers produce AUC = 0.5. Each network was classified with an independently simulated state space. *Empirical Classifications* of 1191 empirical networks spanning 18 kinds of systems. Each circle represents a mechanism. Inset numbers are the number of networks classified as that mechanism. If a network was classified as more than one mechanism the resulting pie graph was made with equal splits for each mechanism because we cannot yet confidently assign probabilities to mixture networks as shown in Fig. 4.

The set of $d \left( N_i, N_j \right)$’s across all pairs of networks constitutes a network state space (e.g. Figs. 1A and 3) within which closer networks have more similar structures, function more similarly, and are more likely to have been generated by the same mechanism. To test if a network came from a particular mechanism our classifier simulates networks from that mechanism, with the same number of nodes and either the directed or undirected version of that mechanism depending on whether the network is itself directed. Moreover, it simulates these networks systematically varying the mechanism’s parameter to find the version of that mechanism which most closely resembles the unknown network. Note that only one parameter is varied in the current implementation of this approach in *netcom*, but the approach would conceptually work the same for a mechanism with multiple governing parameters. Then, many networks (the default is 500) are simulated using this parameter that produces networks most like the network being classified. The test works by comparing the average distance each network is from every other network to the average distance from the unknown network to every other network. The p-value associated with this test is then the percent of these average distances that are larger than the unknown network’s average distance. In this way the classifier tests how likely it is for a mechanism to produce networks like the unknown one being classified.

To reproduce our results, see Table S3 for the parameter values we used with the function ‘classify’ in *netcom*. Most notably, we systematically searched 100 parameter values of each mechanism, with each parameter being tested 50 times. The best fitting parameter, which was the value that on average produced networks most like the one being classified, was then used to simulate 500 networks to create a representative null distribution of how similar networks of that best-fitting kind are to each other.

It is important to recognize that distinguishing mechanisms from each other, which we find most striking about network state spaces like Fig. 1A, is not how our classifier works. Methods that distinguish mechanisms from each other typically produce a probability for each mechanism being the true mechanism which altogether sum to one. This assumes that the true mechanism is one of the ones considered and therefore cannot tell if none of the proposed mechanisms reasonably would create any given network. By testing a network against each hypothesized mechanism independently our approach has the advantage of being able to reject every hypothesized mechanism, and test individual mechanisms.

We also note that the parameter values within each mechanism (size of each point in Fig. 1A) vary smoothly in the network state space, suggesting that our approach may be able to both classify and parameterize mechanisms. Indeed, our package *netcom* estimates parameter values using an average of the known network parameters weighted by their distances from the unknown network.

An ROC (Receiver Operating Characteristic) curve judges a classifier by showing the trade-off between true and false positive labels it produces. In the context of inferring network mechanisms, ROC curves quantify how likely a network classified as a particular mechanism is to actually be from that mechanism. The high AUC (Area Under the Curve) values in Fig. 1B indicate that our classifier has both a high true positive rate and a low false positive rate. This confirms our approach can identify when an empirical network was not produced by any of the candidate mechanisms. Note that undirected ER random networks were not classified much better than a random classifier, which would result in AUC = 0.5. This reflects the randomness of their origin. And, while directed ER random networks were more identifiable, directed versions of the mechanisms we tested were not generally more identifiable than their undirected versions (Fig. 1B).

The ROC curves in Fig. 1B were calculated using networks with 20 nodes. To ensure our approach works for networks more generally, we also calculated these curves for networks with 100 nodes and networks with 20 nodes but created through a standardized growth process used to simulate more complicated network evolutions (e.g. Fig. 3). These ROC curves are shown in Fig. S1 and indicate our approach is robust to network size and kind.

## Empirical network classification

We used our classifier (the function ‘classify’ in *netcom*) to test 1284 empirical networks spanning 17 kinds of physical, biological, and social systems (Fig. 1C). While these networks describe a reasonably large and diverse collection of systems, they were collected opportunistically and should not be construed as representing network data more generally in a way that can be used to test the prevalence of any given mechanism (e.g., in contrast to Broido and Clauset’s^2^ analysis of the prevalence of scale-free networks). All 1284 networks we classified are freely available. For sources, see Table S2.

**Figure 2:**
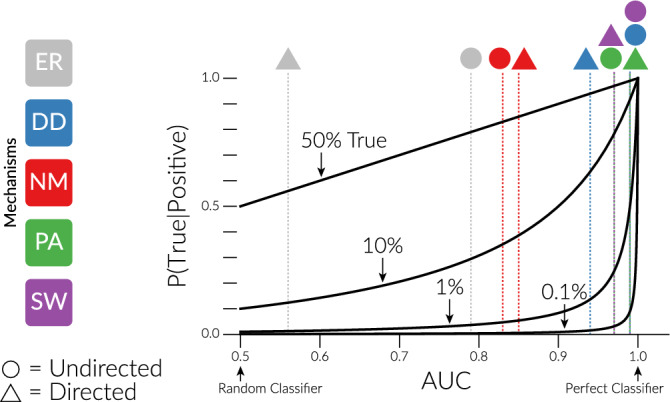
The relationship between the area under an ROC curve (AUC; e.g. Fig. 1B) and the probability that a network classified as a mechanism is actually of that mechanism (i.e. the probability that a positive is a true positive), denoted P(True|Positive). This is calculated using Bayes’ theorem, assuming that AUC represents the accuracy of the test. The four curves show this relationship for different true frequencies of a mechanism: 50%, 10%, 1%, and 0.1%. The vertical lines show the AUC values of our classifier (Fig. 1B) for each of the five mechanisms (color), and as either an undirected or directed process (shape). Stacked lines and shapes indicate identical AUC values.

We found 387 (30.14%) of these empirical networks classified as none of the five mechanisms. Moreover, most of the networks classified as one (or more) of the five mechanisms are likely false positives. Figure 2 shows the relationship between the area under an ROC curve (AUC; e.g. Fig. 1B), and the probability that a positive classification is true using Bayes’ theorem. Fig. 2 assumes that AUC represents the accuracy of the test (technically, it is the average accuracy across all possible p-value cutoffs, but not necessarily the accuracy for positives or negatives at any particular p-value). We cannot know what the true frequency of a mechanism is across all systems, but if we assume all of the networks classified as Small-World are true positives, based on the very high Small-World AUC values in Fig. 1B (0.97 and 0.99), then only 1% of all networks are Small-World (13 purple out of 1284 total in Fig. 1C). With so few true Small-World networks, even a classifier with an AUC value of 0.99 will incorrectly classify networks (false positives) about half of the time (Fig. 2). It therefore seems likely that the majority of the networks classified as random (ER gray; 862 = 67%) and Niche Model (NM red; 113 = 9%) are false positives.

Why do so many of these empirical networks have structural properties unlike any one of the five mechanisms we considered, which have widely been studied as occurring in real systems? One possibility is that these systems are governed by other mechanisms not considered. A second intriguing possibility is that the unclassified systems are governed by a mixture of multiple mechanisms (e.g. 20% preferential attachment and 80% random). This seems all the more reasonable considering that 93 (7.24%) of the networks were classified as plausibly originating from each of multiple mechanisms. However, for many of these 93 networks, the sets of mechanisms contain ER, which our classifier is least sensitive to (and similarly to a lesser extent with NM), so these may be false positives.

To our knowledge there is practically no research on the identifiability of mixtures of mechanisms, let alone their prevalence or function. Moreover, classifying a network as a mixture of mechanisms is not the same as classifying it as more than one mechanism. The latter implies that the network has structures, and possibly functions, like networks governed by each network on its own. To classify a network as a mixture of mechanisms we must first simulate networks that are themselves governed by mixtures of mechanisms which can then be compared to the network of interest.

## Mixture mechanism identifiability

Mixture networks could appear enough like those from a single mechanism to be studied as one, but the mixture would bias parameter estimates. For instance, consider a growing network assumed to be governed by Preferential Attachment, but whose first nodes were actually interacting randomly. Estimates of the preferential power of attachment, which measures inequity in the system, would underestimate how unfair this system actually is. The co-sponsorship network of bills in the US Senate provides a real-world example. Fowler^19^ estimated an attachment power of 6.37—suggesting consensus is rare—but found an attachment power of 1.59 when only considering senators in the same political party. Thus, instead of an overly unfair legislative mechanism governed exclusively by preferential attachment, bill sponsorship may be the result of a more complex and fair intra-party process. Similarly, assuming the internet is governed exclusively by the preferential attachment mechanism, with an estimated attachment power of 2.1^7^, assumes every website links to other websites through an identical process. This is unlikely considering many websites are now made from templates with common media links, acting then in part according to the Duplication and Divergence mechanism. How misleading are these single-mechanism parameter estimates?

**Figure 3:**
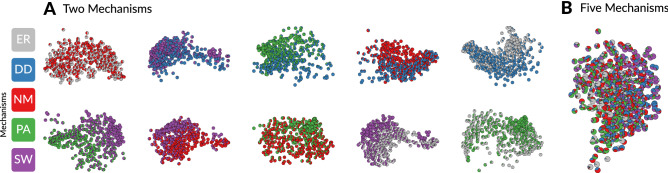
Networks governed by mixtures of Mechanisms. NMDS projections (each with identical axis scaling) of the ensemble distances between networks, as in Fig. 1A, show State Spaces of Mixture Mechanisms. Networks (pies) were made with the ‘make_Mixture’ algorithm in *netcom*. The colors in each pie are proportional to the number of nodes in that network governed by the corresponding mechanism. Mixtures comprised of only *Two Mechanisms* appear identifiable, as a gradient transitioning from one mechanism to the other. However, mixtures with *Five Mechanisms* no longer separate by the proportion of mechanisms in each mixture. These more complex, and perhaps realistic, mixtures are unidentifiable.

**Figure 4:**
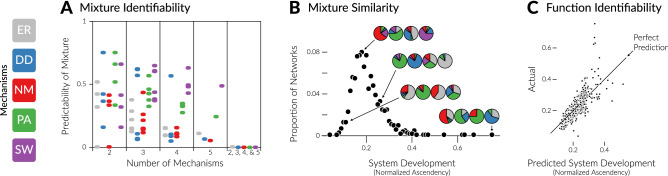
*Mixture Identifiability* Smaller mixtures (e.g. two mechanisms) may be identifiable, but *de novo* identification of mixture mechanisms across larger numbers of mechanisms (e.g. five) or when the number of mechanisms is unknown (far right column) is not possible with our approach. *Mixture Similarity* Different mixtures of mechanisms can produce functionally equivalent systems, here quantified by normalized Ascendency^20^. Pies are example networks generated by mixtures of the five mechanisms, grouped by their shared Ascendency. *Function Identifiability* Leave-one-out cross validation of system development (normalized Ascendency). Each point is a single mixture network, with randomly chosen proportions of the five mechanisms and governing parameters within each mechanism. R^2^ = 0.67.

We are unaware of software to simulate mixture networks, which are needed for training, classifying, and ground-truthing mixture network models. To address this our R library *netcom* includes functions that generate mixture networks in two ways: (i) networks are grown one node at a time each of which attaches to existing nodes according to one mechanism (used in Figs. 3, 4); and (ii) starting with a random fully grown network before, in a random order, iteratively rewiring nodes according to fixed node-specific mechanisms. Both can be simulated, even in the creation of a single network, using the function ‘make_mixture’ in *netcom*. Note that the mixtures used in this study fix each mechanism to be either directed (ER, PA, and NM) or undirected (DD and SW). This allows for systems that have realistically mixed kinds of mechanisms without allowing any particular mechanism to change from directed to undirected interactions within a network.

We find that mixture mechanisms are less identifiable than pure mechanisms. As we can see qualitatively in Fig. 3, and quantitatively in Fig. 4A, as the number of mechanisms in each network increases from two to five, nearby networks in the state space no longer have more similar mixtures of mechanisms than far-apart networks. Our approach seemingly can classify mixtures of only two known mechanisms (Fig. 3A), but we cannot first justify excluding the other three.

The reason we cannot classify arbitrary mixtures of network mechanisms is that different mixtures can produce networks with identical structures. However, all is not lost, because identical networks describe systems that function identically, to the extent that a network can describe a system. Then, even if we cannot recover the mechanisms at play, we can still infer how a system functions. Figure 4 illustrates this with Ascendency^20^. A thermodynamic measure of system growth and development, Ascendency reflects both the quantity of energy flowing through a system (growth) and the proportion of this energy that is cycling within the system instead of being dissipated (development). Whereas our classifier relies on structural properties, Ascendency describes the function of a system. As it was not included in our classification ensemble, Ascendency also provides an out-of-sample test of our ability to classify mixture mechanisms by the way they function, if not their underlying mechanisms.

As Fig. 4B shows, networks governed by different mixtures (proportions of colors in the pie graphs) can have the same Ascendency. However, as seen in Fig. 4C, our approach can be used to accurately predict out-of-sample Ascendency of all sizes and proportions of mixture networks. Thus, even on mixture networks, our approach serves its core purpose of classifying networks according to how they function. Moreover, the non-uniqueness of mixture networks is actually an asset to our approach, making it easier to build a set of candidate models that meaningfully span a functional space within which empirical networks can be classified.

## Conclusions

Knowing the mechanisms that govern a system is key to predicting its behavior in novel conditions. Our results show that we can infer mechanisms from network data by comparing empirical networks to simulated networks across an ensemble of structural properties, with two important caveats.

First, if candidate mechanisms are rarely found in empirical networks, then even sensitive classification approaches, such as ours (Fig. 1B), will have a high false-positive rate (Fig. 2). Our empirical classification results (Fig. 1C) suggest that this may indeed be the case for the five mechanisms we studied.

Second, if systems are governed by different mixtures of mechanisms, these mixtures can be unidentifiable, because multiple different mixtures produce functionally equivalent networks. While this prevents our approach from inferring the proportions of each mechanism at play in such a network, we show that our approach can still predict the way it functions. Ascendency can be directly calculated for any network, but the kind of functional prediction in Fig. 1C may be used to predict functional dynamics that are traditionally observed rather than calculated, like the effects of adding or removing a component (node) of a system (network).

These results illustrate the rich mechanistic information carried in network properties, and the utility of comparative inferences which are becoming more common in network science^21–24^. In thinking about interventions, and exogenous disturbances, we cannot rule out the possibility that functionally identical mixtures would functionally diverge following a structurally significant disturbance and subsequent (re-) growth. This possibility, and mixture networks more generally, merit further study.

Our classifier can work with any network mechanism that can be simulated. This makes it widely useful, but also potentially sensitive to the ways a mechanism is simulated and then compared to a network being classified. As an example, we have tried simulating networks governed by only one mechanism using the tools designed to create mixtures of mechanisms, and found that they are less identifiable (see Fig. S1B with mostly lower AUC values compared to Fig. 1B) than networks of the same kind created using canonical rules that do not all involve system growth. Growing networks itself is a kind of mechanism that imposes structure on any processes occurring through that growth. This makes these networks all more similar to each other. The ways these networks are compared can also affect the sensitivity of the classification. Consider that we only used one set of stack weights to create our ensemble of network properties. The eigenvalues of the first eigenvector of all of these properties across all five mechanisms are informative, but we imagine a more detailed study of the network state space these describe (i.e. Fig. 1A) will meaningfully improve the sensitivity of our general approach. The success we have already had with this approach reflect a deeper truth about learning: the patterns left by a process can be found by comparing observations to a model, but also to other observations.

## Supplementary Information

Supplementary Information 1.

Supplementary Table S1.

Supplementary Table S2.

Supplementary Table S3.

## Data Availability

Sources and metadata for all empirical networks are available in supplementary Table S2. Code is available in the open-source R package *netcom* which can be installed from CRAN^11^ and Github (https://github.com/langendorfr/netcom).
