# Supplementary material for: Empirically classifying network mechanisms
Source: Sci Rep. 2021 Oct 15;11:20501. doi: 10.1038/s41598-021-99251-7 (PMC8519944; doi:10.1038/s41598-021-99251-7)
Supplement: Supplementary file 1 — Supplementary Information 1. [file 41598_2021_99251_MOESM1_ESM.pdf]

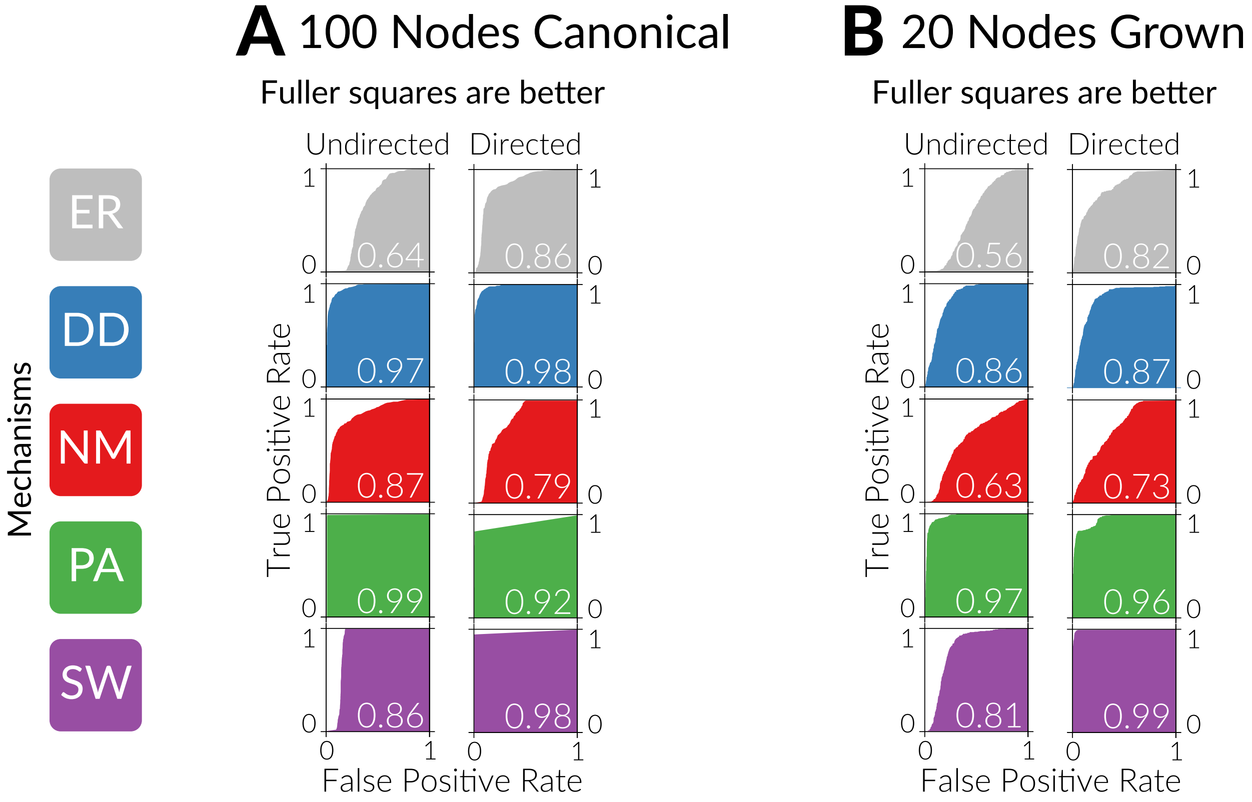


**Figure S1.** **Classification Robustness** ROC curves and corresponding AUC values (inset numbers) for the ability to classify networks simulated differently from the main text Fig. 1. The networks used in Fig. 1 all had 20 nodes whereas in the left panel **A 100 Nodes Canonical**, the networks were simulated identically but with 100 nodes. Note that all empirical networks in this study were limited to those with no more than 100 nodes. The right panel **B 20 Nodes Grown** shows the ROC curves and associated AUC values for networks with 20 nodes but which were grown according to the tools used to make mixture networks but using only a single mechanism each. Together these test our approach's ability to classify networks of different sizes and mechanism implementations. Note that When AUC = 1 the classifier can identify all true positives without including any false positives. Random classifiers produce AUC = 0.5. Each network was classified with an independently simulated state space. Each ROC curve was calculated using 300 networks.

This figure was created by the authors.
